# A ‘movement for improvement’? A qualitative study of the adoption of social movement strategies in the implementation of a quality improvement campaign

**DOI:** 10.1111/1467-9566.12560

**Published:** 2017-06-21

**Authors:** Justin Waring, Amanda Crompton

**Affiliations:** ^1^ Centre for Health Innovation Leadership and Learning Nottingham University

**Keywords:** health service organisations, National Health Service (NHS), organisational theory, professions/professionalisation, quality of care

## Abstract

Given the difficulties of implementing ‘top‐down’ quality improvements, health service leaders have turned to methods that empower clinicians to co‐produce ‘bottom‐up’ improvements. This has involved the adoption of strategies and activities associated with social movements, with clinicians encouraged to participate in collective action towards the shared goal of improvement. This paper examines the adoption of social movement methods by hospital managers as a strategy for implementing a quality improvement ‘campaign’. Our case study suggests that, despite the claim of empowering clinicians to develop ‘bottom‐up’ improvements, the use of social movement methods can be more narrowly concerned with engaging clinicians in pre‐determined programmes of ‘top‐down’ change. It finds a prominent role for ‘hybrid’ clinical leaders and other staff representatives in the mobilisation of the campaign, especially for enrolling clinicians in change activities. The work of these ‘hybrids’ suggests some degree of creative mediation between clinical and managerial interests, but more often alignment with the aspirations of management. The study raises questions about the translation of social movement's theories as a strategy for managing change and re‐inventing professionalism.

## Introduction

In recent years, a variety of methods have been used to improve the quality and safety of healthcare services (Shojania and Grimshaw 2005, Waring *et al*. 2016). Many are informed by techniques found in other industries, and championed by agencies, such as the US Institute for Healthcare Improvement (IHI), the Canadian Foundation for Health Improvement, and the UK Health Foundation. Although there have been breakthroughs, research shows improvements are often marginal and not easily replicated (Dixon‐Woods *et al*. 2012). Critics suggest improvement methodologies are often borrowed naïvely from settings that have limited congruity with healthcare, and attempts to ‘manage’ quality are often resisted by professionals (Waring *et al*. 2016).

In light of these challenges, policymakers have turned to more ‘collaborative’ methods, where clinicians co‐produce ‘bottom‐up’ quality improvement, such as the IHI's ‘Breakthrough Collaboratives’ (Aveling *et al*. 2012, Øvretveit *et al*. 2002). From a sociological perspective, this ‘collaborative turn’ is significant because it re‐invents, rather than reduces healthcare professionalism (Martin *et al*. 2015); as clinicians are empowered to make improvements, albeit in line with policy expectations.

As part of this ‘turn’, improvement advocates have shown interest in the types of engagement and empowerment associated with social movements (Bate *et al*. 2004, del Castillo *et al*. 2016). Social movements are typically described as a form of collective action concerned with influencing social or political institutions (Crossley 2002, Jasper 2010). Social movements are appealing because they provide novel insights into how clinicians can be empowered to implement ‘bottom‐up’ or ‘grassroots’ change (Bate *et al*. 2004). In its broadest sense, our paper is interested in the purposeful adoption of social movement ideas as a means of implementing improvements.

Our specific interest is with how the adoption of social movement ideas could further transform or re‐invent healthcare professionalism, especially through constructing clinical leaders as the ‘flag bearers’ of collective action (Bate *et al*. 2004). Contemporary health reforms often involve health professionals in formal managerial or leadership roles (Waring 2014). These ‘hybrid’ professional‐managers exemplify the re‐invention of professionalism and the emergence of ‘organised professionalism’ (Noordegraaf, 2007). From a sociological perspective, hybrids are often analysed as to whether they promote the collective interests of their profession in a more managed workplace, or advance managerial agendas for the re‐organisation of healthcare work (Numerato *et al*. 2012). More recently, research suggests it is important to look past this dichotomy to understand how hybrids mediate different interests (Gleeson and Knights 2006, Waring and Currie 2009). Relating these debates to the current adoption of social movement methods within healthcare (di Castillo *et al*. 2016), we examine the role of hybrid clinical leaders in the implementation of improvement ‘movements’.

Our paper reports on a qualitative study of the adoption of social movement methods in the implementation of a quality improvement (QI) campaign within an English National Health Service (NHS) hospital. An important caveat is that we do not assume a social movement was necessarily created, nor do we use social movement theories to analyse the observed changes; rather, we are interested in managers’ and hybrid leaders’ purposeful adoption of social movement methods as a strategy for implementing change.

## Social movements for health improvement?

There remains no agreed definition for a social movement. Whilst they share characteristics with both formal political campaigns and informal social trends, they are usually understood as a distinct form of collective action concerned with influencing social or political institutions (Jasper 2010). They are often defined by the shared interests of ‘grassroots’ activists who interact through dense social networks, which can develop over time into more formal campaign organisations (de la Porte and Diani 2006). Drawing on Crossley (2002), social movemets can be conceived as challenging and creatively changing existing authority structures and social and political institutions based upon actors’ shared interests. Although often associated with challenging institutional (e.g. political or corporate) or cultural authority (e.g. the beliefs systems), movements can also have more conservative goals for maintaining social order. Social movements are sociologically interesting because they involve different forms of collective agency for maintaining or changing field‐level institutions (Fligstein and McAdam 2012).

In line with these ideas, social movements have had an influential role in many health reform agendas (Brown and Zavestoski 2004, Levitsky and Banaszak‐Holl 2010), including campaigns for universal healthcare in the US (Hoffman 2010), the promotion of services for marginalised groups (Kitchener 2010) or the introduction of alternative therapies (Goldstein 2010). Movements also oppose reform, such as campaigns against ‘Obamacare’ (Jacobs and Skocpol 2016) or contractual changes for junior doctors in the English NHS (West 2015).

With regards to healthcare quality, social movements have garnered interests because of the potential to engage and empower clinicians in ‘bottom‐up’ change (del Castillo *et al*. 2016). Bate *et al*. (2004: 64) suggest many ‘top‐down’ initiatives struggle to realise improvement because they fail to engage frontline clinicians, whereas a movement approach enables service leaders to leverage the ‘latent potential’ for change and secure ‘wider and deeper participation in a movement for improvement’. The influence of these ideas can be seen with the IHI's ‘5 Million Lives’ campaign, the NHS ‘Sign up for Safety’ campaign, and the Health Foundation's network of ‘Q Fellows’. Recently, the Chief Executive of the English NHS stated ‘every doctor must feel empowered in a social movement’ (cited in del Castillo *et al*. 2016: 20).

The former NHS Modernisation Agency, working in collaboration with Bate and colleagues, has been at the forefront of applying social movement theory to quality improvement (Bate *et al*. 2004, Bate and Robert 2010). They have developed a model for engaging and empowering clinicians in bottom‐up collective action aimed at improving healthcare quality (Bate and Robert 2010). We summarise their three‐stage model to illustrate how social movement theories have informed QI methods.

The first stage describes the need for leaders to ‘frame’ the aspirations or vision for change in ways that aligns individual interests with those of the movement. Framing is a key concept for explaining how movements attract and mobilise members in collective action (Benford and Snow 2000). A ‘collective action frame’ offers a ‘shared understanding of some problematic condition or situation they define as in need of change, make attributions regarding who is to blame, articulate an alternative set of arrangements, and urge others to act in concert to affect change’ (Benford and Snow 2000: 615). For Bate *et al*. (2004) ‘frame alignment’ is an essential first‐step in winning the ‘hearts and minds’ of clinicians.

The second stage involves leaders ‘mobilising’ clinicians in ‘grassroots’ collective action aimed at improvement. Mobilisation is a prominent area of social movement research relating to how collective action is facilitated, how leaders organise activities, and how actors engage with policy processes (Ganz 2013, Morris and Staggenborg 2004). Research suggests that as movements formalise, they develop more formal organisational processes and leadership roles to coordinate collective activities (de la Porte and Diani 2006). For Bate *et al*. (2004) ‘clinical leaders’ can act as ‘flag bearers’ to engage and mobilise clinicians in collective action.

The third stage involves ‘sustaining and mainstreaming’ the changes brought about through collective action. The social movement literature suggests sustainability can occur through influence within political processes, for example, changing laws or attitudes, or becoming a formal organisation or political party (Hensby *et al*. 2011, Meyer and Tarrow 1998). Although, many social movements struggle to make a lasting impact and dissolve into localised interest groups. In the healthcare context, it becomes important to institutionalise the goal of improvement within the cultures of clinical work (Bate *et al*. 2004).

Service leaders’ attraction to social movement ideas is motivated by their commitment to improving healthcare quality, especially through empowering clinicians and changing cultures (Bate *et al*. 2004; del Castillo *et al*. 2016). That said, the adoption of these ideas, like other forms of change management, can be interpreted more critically, especially as re‐aligning clinicians’ interests to overcome resistance (McDonald 2004; Waring and Currie 2009). We relate this more critical line of thinking to contemporary sociological debates on the role of clinical leaders in the re‐organisation of healthcare work.

Returning to the social movement literature, the role of leaders in mobilising collective action is significant (Goodwin and Jasper 2014). According to Ganz (2013), leaders need to frame issues, build relationships, devise strategies and catalyse action. Zald (2005) distinguishes between more senior leaders who determine the ‘priorities’ for change and middle‐level leaders who identify ‘possibilities’ for change. Whilst, Wallace and Schneller (2008) see movement leaders as ‘orchestrating’ change in the space between formal leaders (who set the vision) and frontline managers (who deliver the vision).

A more critical interpretation sees leaders not as representing and empowering grassroots communities, but rather as imposing particular interests to manipulate and steer community action. This can be seen in Pender's (2002) analysis of the World Bank's *Poverty Reduction Strategy*, which claimed to be informed by the experience of over 60,000 people from 60 countries. Despite asserting to empower local voices, Pender suggests its recommendations were largely pre‐determined by senior World Bank officials, with local voices used to justify ‘top‐down’ policy. A similar critique is offered in Frawley's (2015) analysis of the *Action for Happiness* movement, which shows how prominent national figures imposed aspirations for change onto local communities. In such cases, politicisation is far from ‘bottom‐up’ but orchestrated by senior advisors. Such studies suggest the strategies used by social movements to challenge social or political authority, can also be used by those already in authority to manipulate collective action in ways that gives the impression of empowerment (Frawley 2015).

Relating these debates to quality improvement, clinical leaders are described as essential to framing and mobilising collective action (Bate *et al*. 2004; del Castillo *et al*. 2016). More broadly, clinical leadership has emerged as a prominent discourse of contemporary health reform (Martin and Learmonth 2012), because leaders, unlike managers, are embedded in frontline care and better positioned to motivate and empower clinicians (West *et al*. 2014). The role of clinical leaders relates to longstanding sociological debates on professional ‘elites’ and ‘hybrids’ within the social organisation of healthcare work (Waring 2014). These elites, leaders and hybrids have been interpreted as either protecting the collective interests of their profession in a managed workplace (Freidson 1984), or promoting the interests of managers over their clinical colleagues (Numerato *et al*. 2012). Seen from this perspective, the position of clinical leaders within healthcare social movements could be interpreted as either promoting the shared interests of clinicians in forms of ‘bottom‐up’ action, or re‐aligning the interests of clinicians to reflect the ‘top‐down’ aspirations of management. Looking beyond this dichotomy, clinical leaders are increasingly seen as mediating the interests of both management and their profession resulting in novel forms of work organisation (Waring and Currie 2009). Informed by these debates, our study investigated hospital managers’ purposeful adoption of social movement ideas in the implementation a quality improvement (QI) strategy. We asked whether efforts to build a ‘movement for improvement’ was necessarily concerned with fostering ‘bottom‐up’ change, or enrolling clinicians within ‘top‐down’ change.

## The study

### The case study

The research involved a single in‐depth qualitative case study of the adoption of social movement methods within one acute hospital in the English NHS. Following Yin (2013), case study research affords detailed empirical analysis of an exemplary case from which explanatory interpretations can be developed. The case study hospital was identified following a scoping review of hospital QI projects across the English Midlands, involving desk‐based analysis of strategies and interviews with strategic leaders. Three hospitals described their initiatives in terms of a ‘campaign’ or ‘movement’ from which the case study hospital was selected.

The case study was a medium‐sized ‘general’ case study hospital with around 500 beds, including medical, surgical, emergency and maternity services. Between 2013 and 2015 hospital managers introduced a new QI strategy in line with national recommendations, following the inquiry into sub‐standard care at Mid‐Staffordshire NHS Trust (Francis 2013). The main interventions included: (i) a risk control method called ‘stop the line’ (Sugimori *et al*. 1977); (ii) improvement cycles based upon plan‐do‐study‐act (PDSA) methods (Walley and Gowland 2004); and (iii) a revised incident reporting and learning system (Barach and Small 2000). These were supported by (iv) a ‘leadership development’ programme; and (v) a ‘culture change’ initiative (West *et al*. 2014).

**Figure 1:**
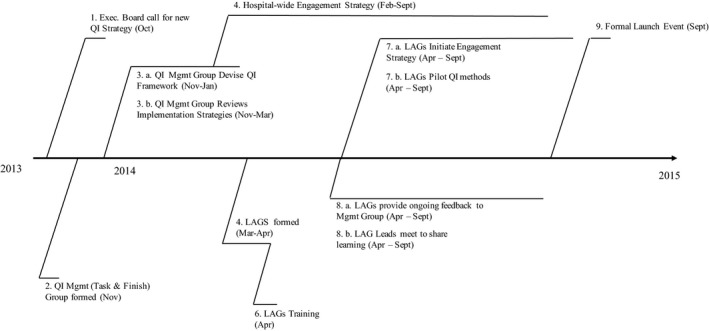
Case study time line

Significantly, the implementation of the QI strategy was informed by senior managers’ interest in social movements, which was the primary focus of our study. As an introduction to the case, in November 2013 a senior ‘leadership team’ was formed to develop the QI strategy, and the study commenced qualitative data collection early 2014. At this time, clinical ‘champions’ and ‘local action groups’ were established across hospital departments, and over six months, various promotional activities were introduced to ‘build the movement’ (Director of Nursing). A ‘go live’ day took place in September 2014 (see Figure 1).

### Data Collection

The study developed an ethnographic account of the adoption and use of social movement ideas within the hospital. Data collection was carried out over 12 months involving non‐participant observations, semi‐structured interviews, focus groups and documentary analysis. An initial set of eleven interviews was carried out with senior managers (4), senior medical and nursing leaders (2), quality and safety managers (2) and senior human resources, communications and operations managers (3). During this time, over 90 hours of observations were undertaken in management offices, team briefings, and training events, which were recorded in handwritten field journals. A second phase of data collection involved 23 interviews with local action group representatives, including, six ‘campaign leaders’, six nurses, four doctors, three departmental managers, and four support workers. Finally, three focus groups were undertaken with staff groups not directly involved in the action groups to understand staff experiences of the implementation processes, including medical representatives (6), nursing representatives (10), allied health professionals (4) and support staff (5). All interviews and focus groups were recorded and transcribed verbatim. The research received ethical approval through university ethics committee.

### Data Analysis

Qualitative data were analysed following an interpretative approach with all authors coding data to establish an agreed coding strategy, with regular meetings to review coding and internal consistency (Corbin and Strauss 2014). Through this process coded data were analysed to develop a descriptive overview of the case study, together with explanatory themes related to the wider theories and debates. The findings develop an account of how social movement ideas were adopted and utilised in the implementation of the QI portfolio; including, managers’ rationale for adopting a social movement approach; the organisation of the ‘campaign’ through the enrolment of clinical leaders, and the reactions of clinicians. Bearing in mind the wider sociological debates on professional‐managerial hybrids, analysis considered the mediating role of clinical leaders in the change process.

## Findings

### Adopting a movement approach

In October 2013, the hospital's executive board established a senior management ‘task‐and‐finish’ group to develop and implement a new QI strategy. This was led by the nursing director, and involved the senior managers for human resources, communications, and quality & safety, and the periodic involvement of the Medical Director and other senior managers. It was described how this group identified relatively quickly (e.g. 2‐3 months) the QI methods to be introduced across the hospital, including incident reporting, PDSA and ‘stop the line’, which was informed by the work of leading improvement agencies (e.g. IHI) and exemplar hospitals (e.g. Virginia Mason in Seattle).

PDSA is a well‐established industry technique. We had elements of it already, but it probably wasn't as thorough as you might expect. So we wanted to do a big push, get it in every department as part of their local improvement work. (Quality manager)

[T]hese techniques make a difference to how people work and patients are treated. We didn't need to re‐invent the wheel for how we improve quality, but we did need to think of different ways to engage staff. (HR manager)

Of interest to our study, the management group described spending more time developing the corresponding implementation strategy. A recurring issue was the need to overcome ‘change fatigue’ stemming from persistent reforms. It was believed many ‘pockets’ of clinicians were resistant to change, as demonstrated by a series of ‘failed’ initiatives, including a new IT system and hospital re‐organisation. Managers described how changing the culture was a pressing challenge.

The doctors can be awkward … They can put the brakes on almost anything. It doesn't matter what we are trying to do. We are seen as the enemy…. some doctors are really engaged, but there are some entrenched views. (Field record with operations manager)

If they see change as not in the interests of their patients or their colleagues, or maybe their service, they simply won't engage. They will come up with all manner of good reasons and talk about the need for evidence, but really it's because they don't like the idea of ‘the hospital’ managing their work … So we need to make them feel it's not about ‘us and them’. It's them. (nursing director)

You can get all the governance structures you like, and that's almost the easy bit, but it's how do you create that governance mind‐set, to ensure behaviours match up with what people should be doing and what's written down. So we started to talk about putting together some sort of development programme, we started to talk about it as ‘boardroom to ward’ kind of approach. (medical director)

Following a review of implementation methods, including incentive schemes and social marketing, the senior management group described their shared interest in social movements. The Medical Director was especially supportive, making references to the ‘Arab Spring’, the community politics of US President Barack Obama, and a seminar attended at Harvard University. These ideas were endorsed by other senior managers who talked of similar ideas advocated by NHS leaders, including the former NHS Modernisation Agency.

We looked at a number of options. I think [communications manager] was really interested in social marketing campaigns. You know, the healthy eating campaigns and things like that? Something about it being viral. (quality manager)

You see something different happening around the world. People are connecting in ways so they work around government to make change happen. If you look at what has happened in North Africa, no one would have imagined … we have been looking at the Obama campaign and the work of Marshall Ganz on social movements. (medical director)

The management group's attraction to social movement ideas seemed to be based on four viewpoints. First, social movements were associated with novel forms of communication and engagement that ‘worked around existing systems’, such as the use of social media and informal networks. Second, social movements were seen as leading to more gradual and impactful change that worked outside of conventional processes. Third, social movements were seen as empowering clinicians to lead change. And fourth, social movements were seen as persuading and influencing ‘difficult people’, rather than forcing change upon resistant groups.

There is a lot we can learn in the way movements reach out to people, they make use of technologies so, you know, governments or whoever don't really see change happening. (director of communications)

What movements show you is that change can happen through people working together, on the street or in the square, and before you know it there is unbelievable change. (medical director)

Campaign groups seem capable of getting people involved. They tap into the emotions and things people care about. (HR manager)

The campaign approach is very popular. The ‘Sign‐up for Safety’ Campaign and the ‘Wash your Hands’ campaign. They communicate with people in ways that helps them see it is really up to them, they can make the difference.’ (nursing director)

We are trying to create a social movement so every member of the workforce sees themselves as responsible and able to make a difference. (quality manager)

One leader summarised the appeal of social movements as a new ‘social contract’ with the clinical workforce, based on the idea of sharing responsibility for change.

We want to create a new social contract with the clinical workforce … not based on the usual forms of management but upon the shared commitment of everybody in the organization. (medical director)

Social movements seemed especially appealing because they gave the impression of change being emergent or disruptive, but also because it could be managed.

If you don't have disruption you don't get change, you have to disrupt to create innovation in my opinion. That's what we are trying to do, to manage disruption in the right way. (quality manager)

You've got to balance autonomy and control, haven't you, because if its complete top‐down control you starve innovation and if its complete freedom you've got chaos. (medical director)

Yet, our observations suggested senior managers had little expectation that frontline clinicians would influence the design of the QI framework, rather their plan was concerned with securing staff participation in the pre‐determined interventions.

It is a movement, but it is a movement with clearly defined objectives and they are about getting the new strategy up and running by next Summer. We have got to get everybody on board, and that means getting them involved. (quality manager)

### Mobilising the movement through local action groups

Senior managers described themselves as relatively detached from frontline practice, and when developing their implementation plans they looked closely at the ‘community building’ and ‘networking’ aspects of social movements. Influenced by prominent guidance (Bate and Robert 2010) they established ‘local action groups’ (LAGs) across the hospital to ‘build the movement’.

The whole thing is a way for us to unlock the bureaucracy that holds us back … information gets lost in translation as it gets passed from managers to frontline staff. The idea is that the movement might go some way to getting information out there and closing the gap between them and us. (nursing director)

The key is infection, to make people talk about it, so they become your sales force … it's like pyramid selling, you infect people with something that excites them and energises them to the extent where they want to go and spill it out to someone else. (communications manager)

Each LAG was comprised of three‐to‐six people, typically drawn from the same hospital department. Although LAG teams had a variety of occupational backgrounds, all were led by established departmental clinical leaders. The initial five LAGs were formed in areas that senior managers regarded as proactive in QI, such as anaesthesia and maternity, with the expectation these would act as ‘role‐models for the rest of the hospital’. This also suggests managers were recruiting clinicians already ‘converted’ to the improvement agenda.

It involved going out there, going into offices and wards and saying, I want you to be a champion, I want you to be a champion, please will you be a champion, can you meet me in my office at twelve o'clock and I'll tell you what you've got to do. (nursing director)

The clinical leads in these areas are supportive and if we get the clinical leads saying ‘we are backing this and it's a good thing’ everyone else will start to realise they should join in’ (field note)

Shortly after selection, a one‐day training workshop was organised for the LAGs. At this event, the QI framework was introduced and the movement‐inspired implementation strategy was outlined. Although most participants seemed familiar with the QI interventions, in part because of their pre‐existing work in this area, they were less familiar with the social movement approach.

It took a bit of education with some of the doctors and one who is now a group leader was very much anti the movement approach. I spoke with him last night and he said, you would never believe that I am now the lead … that's my bonus, getting people like that to be attracted to a be a promoter. (QI trainer)

I was really taken with the communication strategy. It's just so different to what we have tried before. [Nursing director] made a good point that we need to do something different, we have got to shake things up, and be unconventional.’(LAG, ward manager)

In the following three‐month period, the LAGs used different engagement activities to build staff interest in the QI framework. One of the main roles was to translate and communicate the vision for change as set out by hospital managers, making it relevant to frontline staff. Our observations with senior managers found they often communicated in more general terms, eluding to the problems at Mid‐Staffordshire (Francis 2013) or demands of government regulation. In other words, the external push for change. In contrast, when LAG members interacted with clinicians they conveyed a more specific vision, focusing on quality issues relevant to the specific department. In other words, the internal pull for change.

We can see what bothers colleagues. We work with them every day, and we care about the same things. That makes it easier for us to appreciate the benefits of what the Trust is trying to do. (LAG member)

It makes it real. To talk about some event or issue that we know annoyed everyone. That way we can show how things like Stop the Line are going to help make sure it is less likely to happen again. (LAG member)

LAGs were instrumental in implementing a range of campaign‐based initiatives. This included making presentations at departmental meetings, organising training and providing hands‐on guidance in the QI interventions. The approach taken was described as informing and empowering staff, rather than being directive.

We take the position that the manager and clinical directors aren't there to tell everyone what to do, they are servants of everyone. We want to provide support for the initiatives, we aren't interested in telling people what to do, they've got freedom within the system. (LAG leader)

It was a clear picture of ‘we are not doing this to you, you're going to do this yourself, and we are going to give you the tools, the methods and people to deliver it’. (LAG member)

In line with the idea of empowering staff, LAGs often talked of the need for frontline staff to determine how best to use the pre‐defined QI techniques within ‘their’ department, encouraging clinicians to ‘try it out’ and ‘see what works for you’. But here we also noticed an inherent issue with the need to control local action.

We want everybody to be open to new ways. Everyone to be open to learn from each other. So that we're all role models into each other. (LAG member)

Our biggest challenge is to try and coach and control, otherwise we could lose our message very quickly. (LAG leaders)

The LAGs also used novel, more oblique engagement strategies. These included a ‘whisper’ or ‘rumour’ campaign, which senior managers devised through their analysis of social marketing campaigns. LAG leaders described, for example, leaving documents in meeting rooms with the intention of surreptitiously spreading information, gossiping with staff about forthcoming events, and leaking ‘secrets’ about hospital policy. The idea being to build staff members desire to be ‘part of the secret’.

We called it a whisper campaign. For four weeks we did something different. We dropped merchandise out or we'd send poems about the initiatives or quizzes or flyers, and staff were walking around asking ‘what's this all about’. And we'd say if you want to find out, go and find a champion and get a gift bag. (nursing director).

It was a bit sneaky if you think about it. But that's what got people interested. We weren't just issuing another policy … we were trying to get people to ask us for more information. (nursing representative).

The LAGs also championed a hospital‐wide ‘pledge campaign’, encouraging staff to declare support for quality improvement. They also set up information stalls with posters and leaflets promoting the evidence and contribution of the QI framework.

They were a bit like the people in the street who ask you to donate or complete a survey. They went from ward to ward asking people to sign‐up. (ward nurse)

We put leaflets and materials at different sites across the hospital, you know, to build a kind of presence so that everyone knew about it. We asked everyone to show their support by signing up. It was a bit like a petition. (LAG member)

The senior management team encouraged LAGs to champion local initiatives that supported the implementation strategy. For example, frontline staff produced a newsletter about their journey of trialling the QI interventions and some wards hosted ‘safety’ parties. These activities were interesting because they were indirectly concerned with the QI agenda, and appeared more directly concerned with involving people in something ‘fun’ (5).

It was a brilliant day. We had a street party in the reception areas, games and prizes in the lounge. It was something to be really proud of. (LAG representative)

We wanted it to be a campaign, if you like. And fun as well, it wasn't like, this is serious, this is risk and you all need to know…We didn't push the quality improvement angle, we just hoped that would come through. What we wanted was for staff to be interested and engaged. (nursing director)

Although LAGs encouraged local initiatives, this did not necessarily equate to local autonomy. During fortnightly senior management meetings, we observed how LAG leaders were expected to provide updates on local activities, including the number of staff ‘signed‐up’ and progress in implementing QI interventions. It was expected that LAG leaders would seek approval from senior managers for any new initiatives, especially if they had resource implications. It was also observed how LAG leaders rarely advocated the interests of frontline clinicians, for example, where staff were frustrated with the QI framework.

### Clinicians’ reactions

We found frontline clinicians were largely supportive of the QI interventions, in principle, with many regarding incident reporting and audit cycles as expected features of healthcare organisations. Although some had reservations about the purported benefits, there was little evidence of the scepticism anticipated by managers. This could suggest that clinicians were more accepting of QI methods than the literature suggests, or the movement‐inspired implementation strategy had been successful. Indeed, many focus group participants were enthused by the campaign approach.

The introduction event was very dramatic and this was all quite alien to us, but the razzamatazz was important because it meant that the principles behind it and how it fits with patient safety was in your face. (doctor)

What this movement has achieved in my opinion is it has articulated the ‘moral compass’ of what the NHS should do concerning patient safety. It has brought it into the hearts and minds of all of us and not just those with risk management responsibilities. (ward sister)

The study found support for the QI framework, and movement approach, was greatest in those departments that worked closely with LAGs. With the whisper campaign, for example, they seemed to see themselves as party to the ‘secret’. This suggests that LAGs had s strong influence on clinical groups with whom they worked closest.

We felt like we were getting advanced noticed of the things coming down the line … it was quite exciting. (operating nurse)

It's part of culture really. We are always looking to do things better. Good isn't good enough, and all that … We are quite research active … We were one of the first to get involved in the campaign. (unit manager, maternity)

In other areas of the hospital, clinicians appeared more sceptical, especially about the underlying intent of senior management, rather than about the QI techniques themselves. For example, focus groups participants representing surgery, acute medicine and elderly care described the campaign approach as a gimmick, and engagement activities were seen as distracting or superficial. These more critical views came from doctors, nurses and other staff groups working in those departments with limited involvement with LAGs. In direct contrast to the views of one focus group participant described above, another participant questioned the perceived ‘razzmatazz’:

There has been a certain razzmatazz associated with [it], that reflects the management style, but sometimes that gets lost in translation. (doctor)

It just seemed like a lot of rubbish. Spin. We were being sold something but made to think it was our idea. (nurse)

What do they think they are doing? They are getting paid to organise parties and festivals. That's taking well‐paid clinicians away from what they should be doing, which is caring for their patients. It just seems like utter madness. (doctor)

## Discussion

Our study investigated the use of social movement methods in the implementation of a QI portfolio. We did not assume that a social movement was emerging in any objective sense, rather we were interested in managers’ adoption of these ideas as a part of their implementation strategy. In line with our theoretical interests, we wanted to understand whether this ‘movement’ approach necessarily produced the types of ‘bottom‐up’ change proposed by advocates (Bate and Robert 2010), and to examine the role played by ‘hybrid’ professional‐managers.

Our study found managers’ attraction to social movements was shaped, to a large extent, by past difficulties engaging clinicians in organisational change. The novel forms of communication and engagement associated with movements were seen as ways to attract and secure clinical participation. Compared to social movements in other social contexts, however, there was little evidence that managers wanted to foster the type of ‘grassroots’ collective action commonly associated with social movements, nor the ‘bottom‐up’ change anticipated by QI advocates (Bate *et al*. 2004). Rather, managers’ ambitions focused somewhat narrowly on changing local practices to the extent they were receptive to and align with the pre‐defined QI framework. Where managers encouraged local action, this was often limited to piloting interventions or undertaking ‘approved’ implementation activities. It could be argued, therefore, that managers’ adoption of a movement approach was an instrumental strategy for creating a ‘receptive context’ (Pettigrew *et al*. 1992) for the implementation of their ‘top‐down’ QI framework.

It would be wrong to suggest all social movements are characterised by radical social change, and it is also the case that many social movements develop formal ‘top‐down’ leadership structures, especially as they become more organised (de la Porte and Diani 2006, Jasper 2010). However, our study does not find evidence of either radical or conservative grassroots action, nor a social movement organisation that has evolved from grassroots activities. Rather it finds more contrived forms of ‘local action’ that were closely coordinated and aligned with managers’ aspirations for change, and where leadership roles aligned with prevailing management hierarchies within the hospital. In no way are we suggesting that clinicians are disinterested in quality improvement, or not inclined to work together to engender change, but our study found little indication that this strategy supported the type of bottom‐up change anticipated by QI advocates. Rather, our study found parallels with Frawley's (2015) analysis of the ‘action for happiness’ movement, in so much that hospital managers seemed to be imposing particular aspirations for improvement upon clinicians, but giving the impression these were emergent.

Developing our analysis, a prominent feature of the ‘social movement discourse’ is the idea that clinical communities are empowered to realise emergent change (Bate *et al*. 2004, del Castillo *et al*. 2016). It might be argued from our study, however, that this is an illusion of empowerment, or empowerment based on narrowly defined parameters (Pender 2002). Taking a Foucauldian perspective, McDonald (2004) offers a similar critique of the healthcare empowerment discourse, suggesting that appeals to autonomy are often conditioned on the internalisation of particular ethical identities that are aligned with managerial expectations. There are also parallels with Harrison and Wood's (1999) concept of ‘manipulated emergence’ which describes the strategy of policymakers to create the impression that policy is formed through stakeholders’ voluntary participation in deliberative process, but where policy outcomes are ultimately aligned with the intent of government. Seen in this way, service leaders’ use of social movement ideas gives the impression of ‘bottom‐up’ change, but it is change designed to reflect the expectations of management.

Our study highlights further a prominent theme of contemporary healthcare reform, where change methodologies are ‘borrowed’ from other disciplines and settings (Dixon‐Woods *et al*. 2012, Waring *et al*. 2016). Social movements have been analysed extensively within political and organisational sociology, and have found expression in many notable social and political contexts, including heath policy (Brown and Zavestoski 2004, Levitsky and Banaszak‐Holl 2010). Yet the translation and adoption of social movement ideas in our study, and arguably by QI leaders more broadly, appears to have resulted in a highly prescriptive and instrumental view of social movements that is primarily interested in their methods of engagement and empowerment, without necessarily considering the ideological intent or socio‐political contexts within which movements usually emerge. By focusing narrowly on the prescriptive ‘tools for change’, service leaders can fail to recognise the specific contexts and contingencies within which innovations are developed, the underlying theories upon which they are based, and the unintended consequences they can have when applied in different settings (Radnor et al 2012).

Our study was particularly interested in the role of ‘hybrid’ professional‐managers in the adoption of a movement approach. Through their involvement in local action groups, ‘hybrid’ clinical leaders were influential in translating hospital managers’ vision for improvement into a form that was relevant to local clinicians, and creating the necessary receptive context for change. For example, where hospital managers developed a ‘general’ frame for change, local leaders crafted a ‘specific’ action frame that attracted local actors (Benford and Snow 2000). To some extent, these findings resonate with Zald's (2005) characterisation of movement leadership, where senior leaders determine the vision for change, and middle‐level leaders identify possibilities for change. However, Zald's analysis seems relevant to more mature ‘social movement organisations’ where leadership roles emerge from and are aligned with the shared interests of grassroots communities. In our study, the role of clinical leaders was concerned with creating alignment with managers’ imposed vision, and not necessarily with allowing for grassroots interests to shape the overarching strategy. There was no sense of hybrid leaders orchestrating change in the spaces ‘between’ formal management structures, or enabling frontline clinicians to challenge established authority structures (Wallace and Schneller 2008). It appeared therefore that hybrid clinical leaders were less concerned with challenging prevailing organisational structures and voicing counter‐arguments, as they were with supporting the implementation of the QI framework (Waring 2014).

It seems especially significant that the LAGs were largely mapped onto prevailing management hierarchies, with clinical leaders selected from areas regarded as sympathetic to managers’ ambitions for change. This does not suggest the type of emergent action often associated with social movements, but reinforces the idea of top‐down change being managed through these leaders. In their study of hybrid medical‐managers, McGivern *et al*. (2015) distinguish between ‘incidental’ hybrids, who take up such roles as a temporary obligation, and ‘willing hybrids’ who use these roles as part of their professional career narrative. For our study, LAG leaders resemble ‘willing hybrids’ who align their work with managerial strategies for change, further illustrating a re‐invention of professionalism (Martin *et al*. 2015). It is noteworthy, however, that few LAGs were formed in areas where clinicians were known to be critical of management; perhaps resulting in pockets of change across the hospital, and suggesting an uneven re‐invention of professionalism (see also Waring and Currie 2009).

Echoing recent commentaries, we did find limited evidence of hybrids engaging in forms of ‘creative mediation’ between management and clinical staff (Numerato *et al*. 2012, Waring and Currie 2009). There were instances where clinical leaders appeared to modify and mollify top‐down change, as illustrated through their framing activities and support for local experimentation. They also appeared to give feedback to hospital managers with the aim of securing support for their clinical colleagues; albeit those who aligned with management expectations. As such, the hybrid LAG leaders acted as a conduit for two‐way exchange between managers and frontline clinicians (Llewellyn 2000).

In conclusion, our study does not dispute the potential of social movement theories to inform the implementation of healthcare improvement, or to engender broader changes in health policy and service delivery (Brown and Zavestoski 2004, del Castillo 2016). However, we question how these ideas have been translated into the healthcare arena as a model for quality improvement and, through our study, raise questions about whose interests are served when adopting social movement ideas as a basis for clinician empowerment. Service leaders’ enthusiasm for social movements reflects a broader ‘collaborative turn’ in quality improvement thinking, which aims to counter professional resistance to change through empowering clinicians (Bate *et al*. 2004). This involves a re‐invention of healthcare professionalism, with clinicians taking greater responsibility for quality improvement, but importantly, responsibility in line with the expectations of policymakers or managers (Martin *et al*. 2015). When located in this context, we suggest social movement ideas have been appropriated by policymakers and managers as a prescriptive and normative technology of engagement and empowerment, giving the impression of emergent grassroots change, but where such change is ultimately prescribed and even illusionary (Frawley 2015).
